# Comparative analysis of development and survival of two Natal fruit fly *Ceratitis rosa* Karsch (Diptera, Tephritidae) populations from Kenya and South Africa

**DOI:** 10.3897/zookeys.540.9906

**Published:** 2015-11-26

**Authors:** Chrysantus M. Tanga, Aruna Manrakhan, John-Henry Daneel, Samira A. Mohamed, Khamis Fathiya, Sunday Ekesi

**Affiliations:** 1 International Centre of Insect Physiology and Ecology (icipe), P.O. Box 30772 - 00100, Nairobi, Kenya; 2 Citrus Research International, P.O. Box 28, Nelspruit 1200, South Africa

**Keywords:** *Ceratitis rosa*, comparative demography, developmental thresholds, survivorship

## Abstract

Comparative analysis of development and survivorship of two geographically divergent populations of the Natal fruit fly *Ceratitis rosa* Karsch designated as *Ceratitis rosa* R1 and *Ceratitis rosa* R2 from Kenya and South Africa were studied at seven constant temperatures (10, 15, 20, 25, 30, 33, 35 °C). Temperature range for development and survival of both populations was 15–35 °C. The developmental duration was found to significantly decrease with increasing temperature for *Ceratitis rosa* R1 and *Ceratitis rosa* R2 from both countries. Survivorship of all the immature stages of *Ceratitis rosa* R1 and *Ceratitis rosa* R2 from Kenya was highest over the range of 20–30 °C (87–95%) and lowest at 15 and 35 °C (61–76%). Survivorship of larvae of *Ceratitis rosa* R1 and *Ceratitis rosa* R2 from South Africa was lowest at 35 °C (22%) and 33 °C (0.33%), respectively. Results from temperature summation models showed that *Ceratitis rosa* R2 (egg, larva and pupa) from both countries were better adapted to low temperatures than R1, based on lower developmental threshold. Minimum larval temperature threshold for Kenyan populations were 11.27 °C and 6.34 °C (R1 and R2, respectively) compared to 8.99 °C and 7.74 °C (R1 and R2, respectively) for the South African populations. Total degree-day (DD) accumulation for the Kenyan populations were estimated at 302.75 (*Ceratitis rosa* R1) and 413.53 (*Ceratitis rosa* R2) compared to 287.35 (*Ceratitis rosa* R1) and 344.3 (*Ceratitis rosa* R2) for the South African populations. These results demonstrate that *Ceratitis rosa* R1 and *Ceratitis rosa* R2 from both countries were physiologically distinct in their response to different temperature regimes and support the existence of two genetically distinct populations of *Ceratitis rosa*. It also suggests the need for taxonomic revision of *Ceratitis rosa*, however, additional information on morphological characterization of *Ceratitis rosa* R1 and *Ceratitis rosa* R2 is needed.

## Introduction

Amongst the Afro-tropical group of tephritid fruit flies (Diptera: Tephritidae), *Ceratitis rosa* Karsch is considered a serious pest of cultivated fruit (White and Elson-Harris 1992, De Meyer 2001a, Copeland et al. 2006). *Ceratitis rosa* is highly polyphagous being recorded on over 90 species of wild and cultivated crops (De Meyer et al. 2002). In mainland Africa, *Ceratitis rosa* is known only from southern and eastern Africa, being absent from the western and central parts of the continent (De Meyer 2001a). Outside this native range, *Ceratitis rosa* has also been reported from the Indian Ocean islands of Mauritius and La Réunion where it is regarded as a major pest of quarantine importance (Orian and Moutia 1960, De Meyer 2001b, White et al. 2001, Duyck and Quilici 2002). The phytophagous nature of *Ceratitis rosa* and its ability to expand its distribution beyond its native range raises major concern for the horticulture industry in Africa and beyond (De Meyer et al. 2008, Li et al. 2009, Mwatawala et al. 2009, de Villiers et al. 2012).

In Kenya, *Ceratitis rosa* was originally thought to be restricted to the coastal region (De Meyer 2001a). However, on 7^th^ December 2001, Copeland and Wharton (2006) reported the occurrence of *Ceratitis rosa* from fruit of five indigenous and exotic plants in the central highlands of Kenya at an altitude of 1,533-1,771 m above sea level. Prior to this, there were no records of *Ceratitis rosa* in Central Kenya following several wild fruit collections in that area by Copeland and Wharton (2006), which led the authors to conclude that *Ceratitis rosa* was a recent colonizer of the central highlands of Kenya. Subsequent surveys in the area have led to continuous recovery of the pest from mango (S. Ekesi et al. unpublished data). In South Africa, *Ceratitis rosa* is widely distributed across the country but is either scarce or absent in the drier inland regions (De Meyer 2001a, De Villiers et al. 2013). In a review on the fruit fly fauna of South Africa, Bezzi (1924) reported on the collection of two *Ceratitis rosa* groups: (1) *“Ceratitis rosa”* from the northern parts of South Africa and *“Ceratitis fasciventris”* (formerly *Ceratitis rosa*) from the southern and eastern parts of the country. Reports of *Ceratitis rosa* in different parts of the Western Cape was also claimed by some researchers (Hepburn and Bishop 1954).

*Ceratitis rosa* is morphologically very similar to two other species within the same subgenus *Pterandrus*: *Ceratitis fasciventris* (Bezzi) and *Ceratitis anonae* Graham (De Meyer and Freidberg 2006). The 3 species form a complex known as the FAR complex (Virgilio et al. 2013) and are sexually dimorphic (De Meyer and Freidberg 2006). The males within the FAR complex can be readily separated based on differences on their leg and anepisternal pilosity patterns (De Meyer and Freidberg 2006). For example, morphological comparisons of the two *Ceratitis rosa* clusters: *Ceratitis rosa* R1 and *Ceratitis rosa* R2 showed differences in the shape and ornamentation of the mid-tibia of the males (Virgilio et al. 2013). The males of the two *Ceratitis rosa* groups can be distinguished from each other as follows: The black area of the mid tibia of *Ceratitis rosa* R1 reaches the lateral margins while the black area of the mid tibia of *Ceratitis rosa* R2 does not reach the lateral margins (De Meyer et al. 2015).

Recent genetic analysis has shown that the FAR complex is probably five entities, rather than the three taxonomic species (Virgilio et al. 2013). A neighbor Joining tree from these studies showed that morphospecies of *Ceratitis rosa* and *Ceratitis fasciventris* was represented by two well-supported clusters of populations depicted as R1 and R1 (for *Ceratitis rosa*), and F1 and F2 (for *Ceratitis fasciventris*). The authors recommended a thorough assessment of the different ecological requirements (e.g. host preference, thermal tolerance etc) of the two populations of *Ceratitis rosa* given their huge economic significance. The possibility of two forms of *Ceratitis rosa* was earlier suggested in molecular studies by Barr et al. (2006) who associated the forms with geographical distribution of the pest (South Africa and La Reunion form versus Kenyan form). Moreover, differences in thermal developmental rates between *Ceratitis rosa* from La Reunion and South Africa were found in studies conducted separately in the respective countries (Duyck and Quilici 2002, Grout and Stoltz 2007) leading Grout and Stoltz (2007) to suggest the possibility of existence of two biotypes of *Ceratitis rosa*, one being more cold tolerant than the other.

Temperature is the single most important environmental factor determining development and survival of tephritid fruit flies (Fletcher 1989). Temperature effects on development and stage-specific survival have been shown to influence both the quantity and quality of tephritid fruit flies produced (Vargas et al. 1996, Vargas et al. 1997, Brévault and Quilici 2000, Vargas et al. 2000, Duyck and Quilici 2002, Trudgill et al. 2005, Grout and Stoltz 2007, Rwomushana et al. 2008, Vayssières et al. 2008, Liu and Ye 2009, Salum et al. 2013). Various tephritid species have specific optimal temperature range for development limited by lower and upper thresholds (base temperature and upper limit). Below and above these temperature limits, development does not occur and this can vary both with developmental stage and geographical origin (Honék and Kocourek 1990). Information on the thermal requirements of insect groups forms an important basis in understanding and predicting the geographical distribution of the different insect groups.

Given the recent evidence of existence of the two groups of *Ceratitis rosa*, studies were undertaken separately in Kenya and South Africa, spanning across the geographical distribution of the pest in mainland Africa, to determine the thermal developmental rates and thresholds of the two *Ceratitis rosa* types.

## Materials and methods

### Fruit fly cultures

The colonies of the two *Ceratitis rosa* groups (*Ceratitis rosa* R1 and *Ceratitis rosa* R2, “hereafter referred to as R1 and R2”) from Kenya were established at the Animal Rearing and Containment Unit (ARCU) of the International Centre of Insect Physiology and Ecology (*icipe*), Nairobi, Kenya. The *Ceratitis rosa* R1 colony was started with 93 flies (47 males and 46 females) reared from infested fallen guava fruits collected from a farm in Kibarani, Msambweni district, Kenya (S 04°19.628'; E 039°32.411'; 34 m a.s.l). The *Ceratitis rosa* R2 colony was initially started with 29 individuals (14 males and 15 females) recovered from infested mango fruits collected at a smallholder farm in Kithoka, Imenti North district, Kenya (N 00°05'58.9"; E 037°40'39.5"; 1,425 m a.s.l).

Stock cultures of the South African *Ceratitis rosa* groups came from infested jambos, *Syzygium jambos* L. (Alston), and loquat, *Eriobotrya japonica* (Thunb.) Lindl. Collected, respectively, from the following locations: Nelspruit: S 25° 27' 08.19” E30° 58' 11.27”, approx 612 m) and Pretoria: S25° 45' 13.7” E28° 13' 45”, approx 1,368 m a.s.l). The flies originating from Nelspruit were designated as *Ceratitis rosa* R1 by M. De Meyer (Royal Museum for Central Africa) and those from Pretoria were assigned as *Ceratitis rosa* R2

Procedures for obtaining the wild fruit fly populations from infested fruits in both countries were carried out according to the methodology described by Rwomushana et al. (2008). The larvae of the two *Ceratitis rosa* populations reared from the host fruits in each countries were subsequently transferred to carrot-based artificial diet after two generations on fruits (Ekesi et al. 2007).

On the artificial diet, the two *Ceratitis rosa* populations from each country were reared for 5-8 generations before the start of the experiments. In both countries R1 colony was kept at 28 ± 1 °C, 50 ± 8% RH and photoperiod of L12: D12, while the R2 colony was kept at 23 ± 1 °C, 60 ± 10% RH and photoperiod of L12: D12).

### Egg collection

Newly emerged adults were held in well ventilated Perspex cages (30 cm length x 30 cm width x 30 cm height). The eggs of each *Ceratitis rosa* population were collected by offering ripe fruit domes (fruit skin that has the seed and pulp scooped out) to mature adult flies. Numerous small holes were made on the fruit domes made using pins (0.8 mm diameter) to facilitate oviposition by the adult flies. The eggs were collected within a uniform time interval of 1 h after oviposition using a moistened fine camel’s hair brush

### Effect of temperature on development and survival of eggs, larvae and pupae of *Ceratitis rosa*

*Egg*: Using a fine brush, one hundred (100) eggs were randomly selected, counted and carefully lined on moistened sterilized black cloth, which were thereafter placed on top of ≈ 60 g of diet inside a Petri dish. The Petri dishes were immediately transferred to thermostatically controlled environmental chambers (MIR-554-PE, Sanyo/Panasonic cooled incubators, Japan and modified Conviron CMP3023 incubators, Manitoba, Canada were used in Kenya and South Africa, respectively) set at seven constant temperatures of 10, 15, 20, 25, 30, 33 and 35 °C (± 0.03 °C) and 50 ± 8% RH, 12:12 L:D photoperiod. Duration of egg stage was observed at 6-hourly intervals under a binocular microscope to determine the time and percentage hatch. The start time was taken as the time when the eggs were collected from the mango dome or apple and developmental time and survival for each replicate were estimated. The experiments were replicated 5 to 6 times. The required temperatures inside the incubators were regularly monitored using standard thermo-hygrometers and experiments in which temperatures fluctuated more than ±0.03 °C were discarded and not included in the analysis.

*Larva*: One hundred neonate larvae of ~1 h old were randomly obtained from the fruit fly cultures and carefully transferred to squares (either 1cm^2^ or 2 cm^2^) of filter paper. The square filter paper containing neonate larvae were placed on top of a 150 g carrot-based larval diet in either a Petri dish or a plastic container. The Petri dish or plastic container was then placed in a rectangular plastic rearing container carrying a thin layer (~ 0.5 cm) of sterilized sand at the bottom for pupation and then transferred to the thermostatically controlled environmental chamber. The top of the plastic container was screened with light cloth netting material for ventilation. Larvae fed *ad libitum*, and mature larvae were allowed to freely leave the Petri dish into rectangular plastic containers for pupation. The sand was observed daily for newly formed pupae and puparia were separated from the pupation medium by gentle sifting. Records of larval durations were kept for each *Ceratitis rosa* group at each temperature regime. The experiments were replicated 5 times.

*Pupa*: One hundred newly formed pupae (~ 1 h old) were randomly obtained from the fruit fly cultures kept at the rearing conditions described previously. The pupae used were from larvae kept at the same temperature being studied. Pupae used were placed in Petri dishes (8.6 cm diameter) and transferred into aerated Perspex cages (30 cm x 30 cm x 30 cm) to allow for adult emergence. The cages were monitored on a daily basis for adult emergence and pupal developmental time and survival were recorded. The experiments were replicated 5 times.

### Data analyses

The developmental time and percentage survival of each immature life stage of the two *Ceratitis rosa* groups in each country were compared using a two-way analysis of variance (ANOVA). Prior to analysis, the developmental time data and percentages of survivorship were subjected to [log (x + 1)] and arcsine-square-root transformation [Arcsin square root (x+1)], respectively, to meet the assumption of homogeneity (Sokal and Rohlf 1981). Means were further compared where appropriate, by the Student-Newman-Keuls (SNK) (Steel and Torrie 1980) multiple range tests at α = 0.05.

*Linear model*: The linear model expressed as *r* (*T*) = *a* + *bT* was used to estimate the relationship between relevant temperatures and developmental rate of *Ceratitis rosa*. In this model, *r* is the rate of development [=1/Development time (D) in days], *T* is ambient temperature (°C); intercept (*a*) and slope (*b*) are the model parameters. Thermal constant, *K* (=1/*b*) is the number of degree-days (DDs) or heat units above the threshold needed for completion of a developmental stage. Lower temperature threshold (T_min_) was determined using the inverse slope of the fitted linear regression line as the x-intercept (= - *a/b*), and is the estimated lower temperature at which the rate of development is either zero or no measurable development occurs (Campbell et al. 1974). Campbell et al. (1974), provide statistics for the standard error (SE) of the lower developmental threshold (T_min_) and this was used as follows:


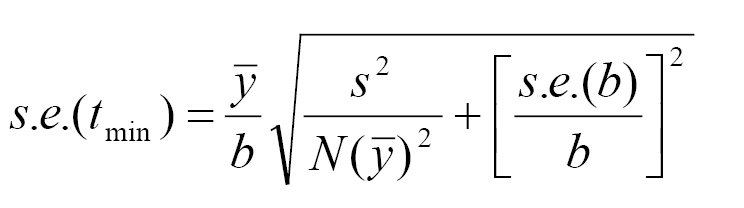


where s^2 ^is the residual mean square of *y*, *y*¯ is the sample mean, and *N* is the sample. Additionally, the size of the SE*_K_* for the thermal constant *K* for the linear model having slope *b* is expressed as:


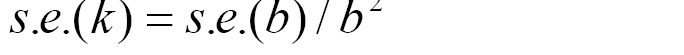


*Nonlinear model*: Several empirical nonlinear models were fitted to the instar specific developmental rate data to estimate the optimum temperature threshold (T_opt_) and upper temperature threshold (T_max_). T_opt_ is the threshold temperature at which developmental rate is maximal, while T_max_ is the lethal threshold at which development ceases. Among the various non-linear models applied to assess the nonlinear relationship, Brière 1 model provided an excellent description of the temperature-dependent development of lowland and highland populations of *Ceratitis rosa* across all temperatures tested for all developmental stages, permitting the estimation of the upper and lower developmental thresholds (Brière et al. 1999). The Brière -1 model is given by the expression:

*r(T)* = *aT* (T – T_min_) × (T_max_ – T)^1/2^

where, *r* is the developmental rate as a function of temperature (T), and *‘a’* is an empirical constant. The following equation from Brière et al. (1999) was used to calculate the optimum temperature:

T_opt _= [4T_max_ + 3T_min_ + (16T_max_^2^ + 9T_min_^2^ – 16T_min_T_max_) ^1/2^]/10

The mean values for T_min_, T_opt_, and T_max_ were determined for each life stage for each group of *Ceratitis rosa* using the results generated by the developmental rate models.

For both the linear and non-linear models, the following statistical items were used to assess the goodness-of-fit: the coefficient of determination (for linear model; R^2^) or the coefficient of nonlinear regression and residual sum of squares (RSS) (for nonlinear models; R^2^). Higher values of R^2^ and lower values for RSS reveal a better fit. For the linear regression, data which deviated from the straight line through the other points were rejected for correct calculation of regression (Campbell et al. 1974).

## Results

### Kenya - Effect of temperature on development of immature life stages

*Egg*: For R1, egg development was longest at 15 °C and shortest at 35 °C (F = 108.2; df = 5, 50; P = 0.0001) (Table 1). For R2, the time required for eggs to hatch decreased from 7.10 ± 0.77 d at 15 °C to 1.83 ± 0.34d at 33 °C (F = 43.25; d.f. = 4, 51; P = 0.0001). There were significant differences in egg developmental duration at 15 °C (F = 9.803; d.f. = 1, 29; P = 0.0040), 20 °C (F = 13.84; d.f. = 1, 21; P = 0.0013) and 30 °C (F = 4.859; d.f. = 1, 17; P = 0.0416) between the two *Ceratitis rosa* groups. However, no significant differences in egg developmental duration were observed between the two *Ceratitis rosa* groups at 25 °C (F = 2.075; d.f. = 1, 15; P = 0.1700) and 30 °C (F = 0.946; d.f. = 1, 12; P = 0.3500). The eggs of both *Ceratitis rosa* groups did not develop at 10 °C. At 35 °C, eggs of R2 also failed to hatch.

**Table 1.** Mean ± SE developmental time (days) of immature stages of *Ceratitis rosa* R1 and *Ceratitis rosa* R2 from Kenya at different constant temperatures.

Temperature (°C)	Egg		Larva		Pupa		Total (days)	
	***Ceratitis rosa* R1**	***Ceratitis rosa* R2**	***Ceratitis rosa* R1**	***Ceratitis rosa* R2**	***Ceratitis rosa R1***	***Ceratitis rosa* R2**	***Ceratitis rosa* R1**	***Ceratitis rosa* R2**
10	-	-	-	-	-	-	-	-
15	8.91 ± 0.51^a^B	7.10 ± 0.77^a^A	28.71 ± 0.65^a^B	23.93 ± 0.64^a^A	32.54 ± 0.85^a^B	27.79 ± 0.64^a^A	68.64 ± 1.79^a^B	58.85 ± 1.39^a^A
20	5.50 ± 0.59^b^B	3.82 ± 0.34^b^A	14.92 ± 0.56^b^B	12.36 ± 0.51^b^A	19.31 ± 0.53^b^B	16.77 ± 0.55^b^A	39.0 ± 0.85^b^B	32.67 ± 0.59^b^A
25	2.43 ± 0.24^c^A	2.90 ± 0.33^bc^A	8.92 ± 0.52^c^B	10.17 ± 0.50^c^A	9.92 ± 0.43^c^B	13.82 ± 0.59^c^A	19.60 ± 1.33^c^B	27.20 ± 0.81^c^A
30	1.90 ± 0.33^c^B	2.56 ± 0.24^bc^A	7.75 ± 0.58^d^B	9.71 ± 0.41^c^A	8.31 ± 0.42^d^B	10.85 ± 0.48^d^A	17.30 ± 1.33^c^B	23.0 ± 0.81^d^A
33	1.50 ± 0.24^c^A	1.83 ± 0.34^c^A	7.36 ± 0.50^d^B	9.36 ± 0.30^c^A	No emergence	No emergence	-	-
35	1.38 ± 0.23^c^B	0.00 ± 0.00A	6.77 ± 0.52^d^B	0.00 ± 0.00A	No emergence	No emergence	-	-

Means in the same column followed by the lower case and in the same row followed by the same upper case letter are not significantly different by Student – Newman – Keul’s (SNK) test, P < 0.05.

*Larva*: At larval stage, the trend was similar to egg with developmental duration decreasing from 28.71 ± 0.65 d at 15 °C to 6.77 ± 0.52 d at 35 °C (F = 705.6; d.f. = 5, 72; P = 0.0001) for R1 and from 23.93 ± 0.64 d at 15 °C to 9.36 ± 0.30 d at 33 °C (F = 422.5; d.f. = 4, 60; P = 0.0001) for R2 (Table 1). There were significant differences in the duration of larval development at all tested temperatures between the two *Ceratitis rosa* groups at 15–35 °C (F = 7.2 – 84.1; d.f. = 1, 25; P = 0.0135 – < 0.0001) (Table 1). At 15 and 20 °C, the larval developmental duration of R1 was significantly longer than that of R2. In contrast, the larval developmental duration of R1 was significantly shorter at 25, 30, 33 and 35 °C compared to R2. The larvae of both *Ceratitis rosa* groups failed to develop at 10 °C. Also at 35 °C no development occurred for the R2.

*Pupa*: At 10, 33 and 35 °C no eclosion was observed for both *Ceratitis rosa* groups (Table 1). Pupal developmental duration of R1 and R2 varied significantly between the other temperatures (F = 455.9; d.f. = 3, 47; P < 0.0001 and F = 945.5; d.f. = 3, 48; P = 0.0001, respectively). Moreover, the pupal developmental duration varied significantly between the two *Ceratitis rosa* groups at 15 °C (F = 54.7; d.f. = 1, 25; P < 0.0001), 20 °C (F = 28.66; d.f. = 1, 24; P < 0.0001), 25 °C (F = 69.64; d.f. = 1, 22; P < 0.0001) and 30 °C (F = 41.09; d.f. = 1, 24; P < 0.0001). The longest pupal developmental duration occurred at 15 °C for both R1 and R2. It took 8.31 ± 0.4 days for R1 and 10.85 ± 0.48 days for R2 to reach eclosion at 30 °C.

*Egg-adult*: Total developmental duration from egg to adult for R1 and R2 was longest at 15 °C and shortest at 30 °C. Significant differences were found between the two *Ceratitis rosa* groups when egg to adult developmental durations were compared across all the temperatures (Table 1) (F = 57.6 – 143.6; d.f. = 1, 25; P = 0.0037 – < 0.0001).

### Kenya - Temperature-dependent developmental rates

Estimated parameter values of the linear and nonlinear models are presented in Table 2. For each *Ceratitis rosa* group, a strong and positive linear relationship was observed between temperature and development rates for egg, larval and pupal stages (Table 2).

**Table 2.** Parameter estimates and their approximate standard errors for linear and Brière-1 nonlinear models describing the relationship between temperature and development rate (1/D) of *Ceratitis rosa* R1 and *Ceratitis rosa* R2 from Kenya.

Model	Parameters	*Ceratitis rosa* R1			*Ceratitis rosa* R2		
		Egg	Larva	Pupa	Egg	Larva	Pupa
Linear	*a*	-0.412	-0.077	-0. 080	-0.270	-0.041	-0. 043
	*b*	0.035	0.008	0.007	0.0263	0.006	0.005
	K	28.57 ± 2.68	133.33 ± 7.24	140.85 ± 33.13	37.04 ± 1.96	172.41 ± 37.75	204.08 ± 30.28
	T_min_	11.77 ± 1.50	10.27 ± 2.54	11.31 ± 2.26	10.0 ± 0.83	7.07 ± 3.99	8.73 ± 2.15
	*RSS*	2.6 × 10^-5^	4.2 × 10^-5^	1.4 × 10^-4^	2.0 × 10^-3^	8.1 × 10^-5^	1.3 × 10^-4^
	R^2^	0.999	0.991	0.936	0.899	0.908	0.895
Brière-1	T_min_	14.23 ± 1.08	11.27 ± 0.71	11.66 ± 0.47	9.66 ± 1.45	6.34 ± 0.84	8.09 ± 0.69
	T_max_	37.0 ± 0.22	37.0 ± 8.71	33.0 ± 1.28	35.0 ± 2.64	35.0 ± 1.19	33.0 ± 1.26
	T_opt_	31.44	30.98	27.87	29.16	28.70	27.35
	R^2^	0.945	0.896	0.835	0.992	0.898	0.905

Using the linear model, the lowest developmental threshold for eggs was estimated at 11.8 °C for R1 and 10.0 °C for R2. The egg stage required 28.57 degree-days (DD) to complete development in the R1 and 37.04 DD in the R2. *Ceratitis rosa* R1 required 133.33 DD above the development threshold of 10.27 °C to complete development from larval stage to the pupal stage while R2 took 172.41 DD to develop above a threshold of 7.07 °C (Table 2). The lower developmental thresholds for pupae of R1 and R2 were calculated as 11.3 and 8.7 °C, respectively. The corresponding thermal constants of the pupal stage were 140.85 DD for R1 and 204.08 DD for R2.

**Figure 1.**
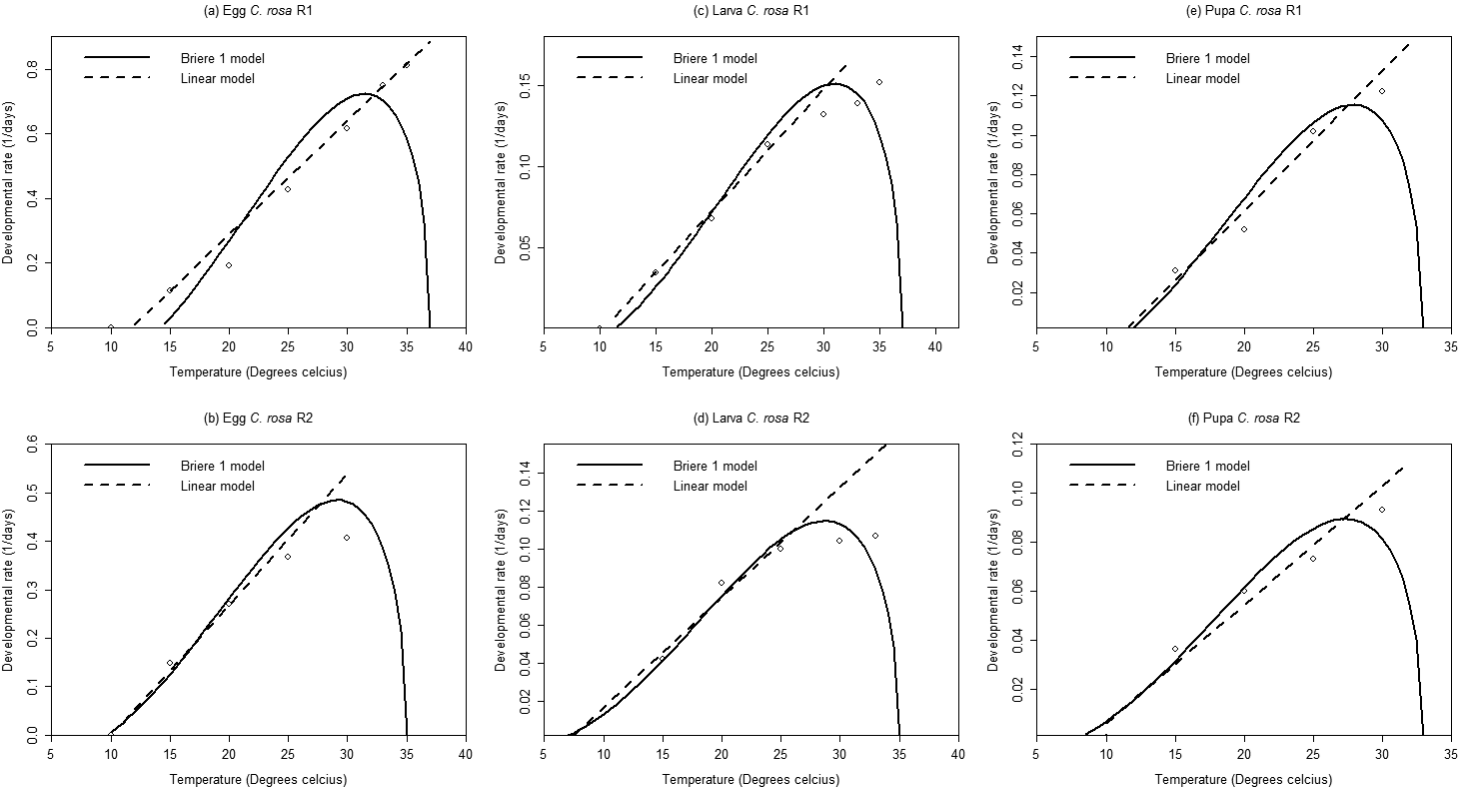
Linear and non-linear regressions of temperature related developmental rates of immature stages of two groups of *Ceratitis rosa* from Kenya.

For R1, the low developmental thresholds generated by the Brière-1 model were found to be slightly higher for egg, larva and pupa compared to those estimated by the linear regression model while for R2 the lower developmental thresholds estimated were slightly lower for egg, larva and pupa (Figure 1 and Table 2). The model estimated optimum temperature range of 27.9–31.4 °C for R1 and 27.4–29.2 °C for R2 (Table 2). The lethal temperatures for R1 and R2 were estimated to range from 33.0–35.0 °C and 33.0–37.0 °C, respectively, for the various developmental stages (Table 2).

### Kenya - Survival of immature stages

At the egg stage, percentage survival ranged from 76.8 ± 4.3% at 35 °C to 93.8 ± 2.0% at 25 °C in R1 (F = 4.75; d.f. = 5, 24; P = 0.0037) and 80.4 ± 3.2% at 33 °C to 91.8 ± 1.8% at 20 °C (F = 5.17; d.f. = 4, 20; P = 0.0050) in R2 (Table 3).

**Table 3.** Mean ± SE survivorship (%) of immature stages of *Ceratitis rosa* R1 and *Ceratitis rosa* R2 from Kenya at different constant temperatures.

Temperature(°C)	Egg		Larva		Pupa	
	*Ceratitis rosa* R1	*Ceratitis rosa* R2	*Ceratitis rosa* R1	*Ceratitis rosa* R2	*Ceratitis rosa* R1	*Ceratitis rosa* R2
10	0.00 ± 0.00^c^	0.00 ± 0.00^c^	0.00 ± 0.00^d^	0.00 ± 0.00^c^	0.00 ± 0.00^c^	0.00 ± 0.00^c^
15	81.6 ± 2.94^ab^A	87.4 ± 1.50^a^A	75.8 ± 2.22^b^A	84.6 ± 4.66^a^A	90.6 ± 2.09^a^A	92.4 ± 2.20^a^A
20	85.8 ± 2.46^ab^A	91.8 ± 1.80^a^A	80.8 ± 3.88^ab^A	86.8 ± 3.09^a^A	91.6 ± 1.44^a^A	95.2 ± 1.16^a^A
25	93.8 ± 2.01^a^A	91.6 ± 1.03^a^A	87.6 ± 1.44^a^A	83.6 ± 2.29^a^A	94.2 ± 1.07^a^A	91.4 ± 1.78^a^A
30	92.2 ± 1.98^a^A	89.4 ± 1.99^a^A	85.4 ± 2.25^ab^A	81.8 ± 2.27^a^A	81.2 ± 2.89^b^A	78.2 ± 3.56^b^A
33	88.2 ± 3.57^ab^A	80.4 ± 3.23^b^A	78.4 ± 1.29^ab^B	67.6 ± 1.63^b^A	0.00 ± 0.00^c^	0.00 ± 0.00^c^
35	76.8 ± 4.26^b^B	0.00 ± 0.00A	60.6 ± 2.96^c^B	0.00 ± 0.00A	0.00 ± 0.00^c^	0.00 ± 0.00^c^

Means in the same column followed by the same lower case and in the same row followed by the same upper case letter are not significantly different by Student – Newman – Keul’s (SNK) test, P < 0.05.

For R1, survival rate was lowest at 35 °C and highest at 25 °C (F = 13.22; d.f. = 5, 24; P < 0.0001) while for R2, survivorship at larval stage ranged between 67.6 ± 1.6% at 33 °C to 86.8 ± 3.1% at 25 °C (F = 5.19; d.f. = 4, 20; P = 0.0049) (Table 3). No significant differences between the two *Ceratitis rosa* groups were observed when larval survival was compared over a range of 15–30 °C except at 33 and 35 °C.

**Table 4.** Mean ± SE developmental time (days) of immature stages of *Ceratitis rosa* R1 and *Ceratitis rosa* R2 from South Africa at different constant temperatures.

Temperature (°C)	Egg		Larva		Pupa		Total days	
	***Ceratitis rosa* R1**	***Ceratitis rosa* R2**	***Ceratitis rosa* R1**	***Ceratitis rosa* R2**	***Ceratitis rosa* R1**	***Ceratitis rosa* R2**	***Ceratitis rosa* R1**	***Ceratitis rosa* R2**
10	-	-	-	-	-	-	-	-
15	7.53 ± 0.10^a^A	7.40 ± 0.06^a^A	17.73 ± 0.19^a^B	20.74 ± 0.40^a^A	36.56 ± 0.28^a^A	36.18 ± 0.29^a^A	61.80 ± 0.24^a^A	64.72 ± 0.45^a^B
20	3.22 ± 0.00^b^A	3.06 ± 0.00^b^A	11.38 ± 0.12^b^B	13.63 ± 0.36^b^A	17.14 ± 0.36^b^A	19.02 ± 0.87^b^A	31.75 ± 0.43^b^A	35.71 ± 1.06^b^B
25	2.14 ± 0.01^c^A	2.11 ± 0.03^d^A	7.59 ± 0.11^d^B	8.44 ± 0.12^d^A	11.81 ± 0.22^c^A	12.09 ± 0.24^c^A	21.54 ± 0.20^c^A	22.67 ± 0.23^c^B
30	1.61 ± 0.00^e^A	1.60 ± 0.02^e^A	7.90 ± 0.03^c^B	9.92 ± 0.61^c^A	7.53 ± 0.02^d^	-	17.04 ± 0.05^d^	-
33	1.69 ± 0.01^e^B	1.99 ± 0.08^d^A	5.74 ± 0.06^e^	6.73*	-	-	-	-
35	1.88 ± 0.03^d^A	2.36 ± 0.03^c^B	6.32 ± 0.05^f^	7.85 ± 0.00**^d^	-	-	-	-

Means in the same column followed by the same lower case and in the same row followed by the same upper case letter are not significantly different by Student – Newman – Keul’s (SNK) test, P < 0.05.

No pupae survived at 10, 33 and 35 °C for both *Ceratitis rosa* groups (Table 3). For R1, survival ranged from 81.2 ± 2.9% at 30 °C to 94.2 ± 1.1% at 25 °C (F = 8.097; d.f. = 3, 16; P = 0.0017). Survival ranged from 78.2 ± 3.6% at 30 °C to 95.2 ± 1.2% at 20 °C in R2 (F = 10.43; d.f. = 3, 16; P = 0.0005). Survival did not differ significantly between the two *Ceratitis rosa* groups at all temperatures.

### South Africa - Effect of temperature on development of immature life stage

*Egg*: The time required for eggs to hatch ranged from 7.53 ± 0.10 d at 15 °C to 1.69 ± 0.01d at 33 °C (F = 1701.32; d.f. = 5, 44; P < 0.0001) for R1. On the other hand, the egg developmental time of R2 was longest at 15 °C and shortest at 30 °C (F = 742.34; d.f. = 5, 46; P < 0.0001). However, no significant differences in egg developmental duration were observed between the two groups of *Ceratitis rosa* at 15, 20, 25, and 30 °C. The eggs of both *Ceratitis rosa* groups failed to develop at 10 °C.

*Larva*: At larval stage, developmental duration was generally shorter for R1 compared to R2 at temperatures ranging from 15 °C to 35 °C (Table 1). The developmental duration of R1 decreased from 17.73 ± 0.19 d at 15 °C to 5.74 ± 0.06 d at 33 °C (F = 1765.82; d.f. = 5, 17; P < 0.0001) while that of R2 decreased from 20.74 ± 0.40 d at 15 °C to 6.73 d at 33 °C (F = 133.58; d.f. = 4, 13; P < 0.0001). The larvae of both *Ceratitis rosa* groups did not develop at 10 °C (Table 4).

*Pupa*: For R1 no eclosion was observed at 10, 33 and 35 °C while for R2, no eclosion was recorded at 10, 30, 33 and 35 °C. Pupal developmental duration of both R1 (F = 2578.64; d.f. = 3, 12; P < 0.0001) and R2 (F = 495.54; d.f. = 2, 9; P < 0.0001) varied significantly when compared across the tested temperatures. Between the two *Ceratitis rosa* groups, again no significant differences in pupal development were observed at 15, 20 and 25 °C (Table 4). The longest pupal developmental duration for R1 was 36.56 ± 0.28 d at 15 °C and that of R2 was 36.18 ± 0.29 d at the same temperature.

*Egg-adult*: Total developmental duration from egg to adult for R1 was longest at 15 °C and shortest at 30 °C. For R2, in contrast, there was no complete development of the immature life stages at 30 °C. Total developmental duration from egg to adult for R2 was longest at 15 °C and shortest at 25 °C. Significant differences were found between the two *Ceratitis rosa* groups when egg to adult developmental durations were compared across all the temperatures (Table 4) (R1: F =179.48, d.f. = 1, 11, P = < 0.0001; R2: F = 669.34, d.f. = 1, 8, P < 0.0001). The egg-adult development of R1 was significantly faster than that of R2 at temperatures ranging from 15 °C to 25 °C (Table 4).

### South Africa - Temperature-dependent developmental rates

Estimated parameter values of the linear and nonlinear models are presented in Table 5. A positive linear relationship was observed between temperature and development rates for egg, larval and pupal stages for both *Ceratitis rosa* groups.

**Table 5.** Parameter estimates and their approximate standard errors for linear and Brière-1 nonlinear models describing the relationship between temperature and development rate (1/D) of *Ceratitis rosa* R1 and *Ceratitis rosa* R2 from South Africa.

Model	Parameters	*Ceratitis rosa* R1			*Ceratitis rosa* R2		
		Egg	Larva	Pupa	Egg	Larva	Pupa
**Linear**	a	-0.469	-0.080	-0.078	-0.323	-0.073	-0.056
	b	0.041	0.009	0.007	0.032	0.008	0.006
	K	24.29 ± 3.29	117.12 ± 9.04	145.94 ± 14.0	31.47 ± 0.89	131.34 ± 10.6	181.49 ± 9.68
	T_min_	11.39 ± 1.51	9.42 ± 0.76	11.44 ± 1.19	10.18 ± 0.34	9.61 ± 0.78	10.15 ± 0.57
	RSS	7.8 × 10^-3^	5.4 × 10^-5^	1.2 × 10^-4^	2.0 × 10^-4^	9.4 × 10^-5^	4.3 × 10^-6^
	R^2^	0.931	0.982	0.973	0.997	0.981	0.994
**Brière-1**	T_min_	12.47 ± 3.11	8.99 ± 2.44	10.97 ± 4.50	9.60 ± 1.65	7.74 ± 4.01	10.47 ± 1.92
	T_max_	36.53 ± 1.05	31.86 ± 6.28	33.0 ± 0.00	36.5 ± 2.64	32.57 ± 1.37	30.0 ±0.00
	T_opt_	30.79	26.57	27.67	30.36	26.96	25.32
	R^2^	0.976	0.993	0.952	0.997	0.997	0.990

For the egg, the lowest developmental threshold was estimated to be 11.39 °C for R1 and 10.18 °C for R2. The egg stage required 24.29 DD to complete development in R1 and 31.47 DD in R2. The R1 group required 117.12 DD to develop above a threshold of 9.42 °C from larval stage to the pupal stage while R2 required 131.34 DD to develop above a threshold of 9.61 °C (Table 5). The lower developmental thresholds for the pupal stages of R1 and R2 were estimated at 11.44 and 10.15 °C, while the corresponding thermal constants were 145.94 DD and 181.49 DD, respectively.

**Figure 2.**
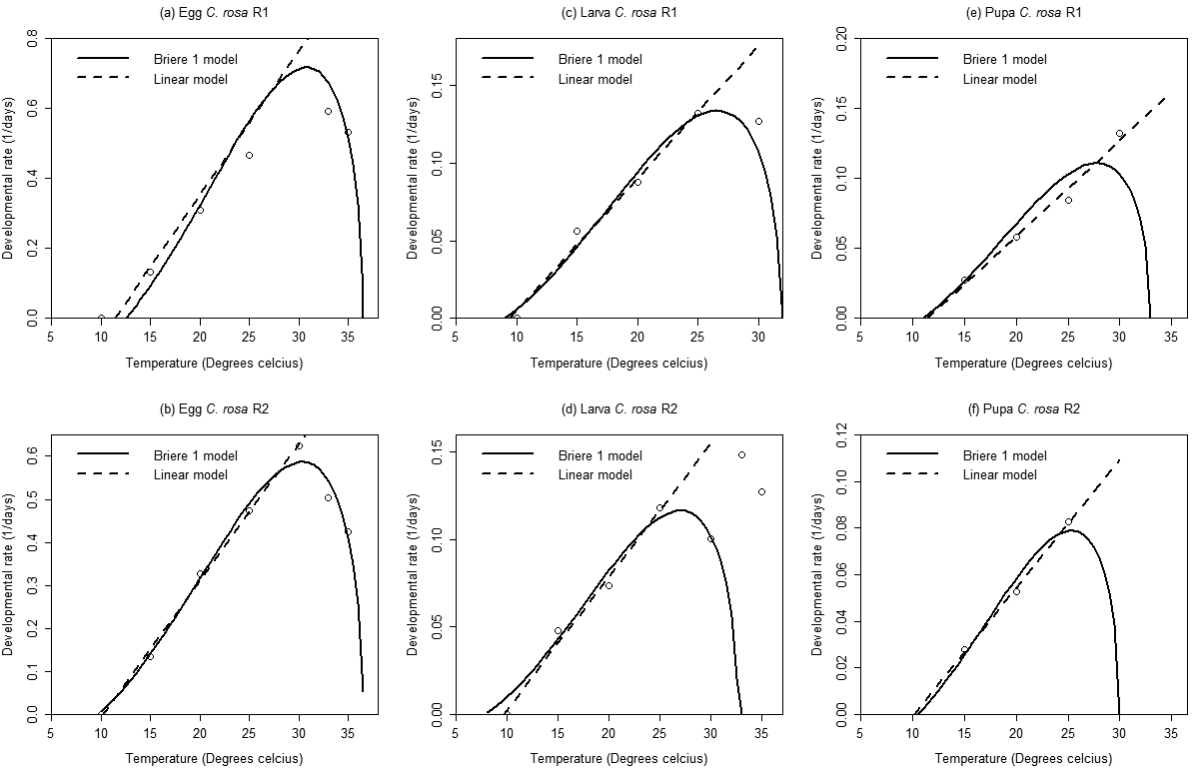
Linear and non-linear regressions of temperature related developmental rates of immature stages of two groups of *Ceratitis rosa* from South Africa

The low developmental threshold values generated by the Brière-1 model for larva and pupa stages for both *Ceratitis rosa* groups were found to be lower compared to values estimated by the linear regression model (Table 5). For R1 the lower developmental thresholds of egg, larva and pupa were slightly different compared to that of R2 (Figure 2 and Table 5). An optimum temperature range of 26.57–30.79 °C was estimated for R1 and 26.96–30.36 °C for R2 for the various developmental stages. The lethal temperatures for R1 and R2 were estimated to range from 31.86–36.53 °C and 30.0–36.5, for the various developmental stages (Table 5).

### Survival of immature life stages

At the egg stage, the percentage survival of R1 (F = 92.63; d.f. = 6, 47; P < 0.0050) was significantly higher compared to that R2 (F = 22.94; d.f. = 6, 49; P = 0.0070) across the temperature range of 15 °C to 35 °C (Table 6). The highest survival rate of R1 was recorded at 20 °C, while that of R2 was recorded at 30 °C.

**Table 6.** Mean ± SE survivorship (%) of immature stages of *Ceratitis rosa* R1 and *Ceratitis rosa* R2 from South Africa at different constant temperatures.

Temperature (°C)	Egg		Larva		Pupa	
	***Ceratitis rosa* R1**	***Ceratitis rosa* R2**	***Ceratitis rosa* R1**	***Ceratitis rosa* R2**	***Ceratitis rosa* R1**	***Ceratitis rosa* R2**
10	0.00 ± 0.00^c^	0.00 ± 0.00^c^	0.00 ± 0.00^e^	0.00 ± 0.00^d^	-	-
15	77.00 ± 4.99^b^A	54.67 ± 8.74^b^B	61.33 ± 4.41^b^A	73.67 ± 2.91^a^A	84.10 ± 3.91^a^A	90.06 ± 1.94^a^A
20	96.00 ± 2.08^a^A	58.00 ± 2.31^ab^B	61.67 ± 4.06^b^A	41.67 ± 1.86^b^B	62.71 ± 5.15^b^A	69.37 ± 6.47^b^A
25	90.67 ± 0.99^a^A	61.33 ± 1.20^ab^B	72.67 ± 1.45^b^A	23.33 ± 2.85^c^B	68.18 ± 8.67^b^A	52.38 ± 9.52^c^A
30	93.22 ± 0.89^a^A	75.11 ± 2.23^a^B	87.00 ± 2.65^a^A	39.33 ± 8.76^b^B	68.33 ± 2.73^b^	0.00 ± 0.00^d^
33	79.50 ± 3.48^b^A	66.80 ± 3.12^ab^B	36.33 ± 4.10^c^A	0.33 ± 0.33^d^B	0.00 ± 0.00^c^	0.00 ± 0.00^d^
35	74.00 ± 1.00^b^A	57.33 ± 2.92^b^B	22.25 ± 3.22^d^A	0.80 ± 0.58^d^B	0.00 ± 0.00^c^	0.00 ± 0.00^d^

Means in the same column followed by the same lower case and in the same row followed by the same upper case letter are not significantly different by Student – Newman – Keul’s (SNK) test, P < 0.05.

For R1, percentage survival of the larval stage ranged between 22.25 ± 3.22% at 35 °C to 87.0 ± 2.65% at 30 °C (F = 77.55; d.f. = 6, 25; P < 0.0001), while that for R2 ranged between 0.33 ± 0.33% at 33 °C to 73.67 ± 2.91% at 15 °C (F = 86.56; d.f. = 6, 22; P < 0.0001). For both *Ceratitis rosa* groups, no significant difference in larval survival was observed at 15 °C. However at temperatures ranging from 20 °C to 35 °C, percentage larval survival of R1 was significantly higher compared to R2 (Table 6).

For R1, no eclosion was recorded at 10, 33, 35 °C while for R2, no pupa survived at 10, 30, 33 and 35 °C. The highest pupal survival rate for both R1 and R2 was recorded at 15 °C. For R1, the lowest survival rate was recorded at 20 °C, while that of R2 was recorded at 25 °C. Percentage pupal survival of R1 (F = 94.25; d.f. = 5, 18; P < 0.0001) and R2 (F = 145.06; d.f. = 5, 18; P < 0.0001) were significantly different when compared across the test temperatures.

## Discussion

The study on the developmental rates of the two parapatric *Ceratitis rosa* groups across the geographical range of the species in question - *Ceratitis rosa* showed different trends according to the area of occurrence. In the north eastern limit of *Ceratitis rosa*, R1 was more heat tolerant and less cold tolerant than R2. In the southern limit of the pest, R1 was more heat tolerant compared to R2 but not necessary less cold tolerant than R2.

Duyck and Quilici (2002) published the first report on development of *Ceratitis rosa* at different constant temperatures. The authors reported that immature stages of *Ceratitis rosa* were unable to complete development at 35 °C and survivorship was also extremely low at 30 °C for all the immature life stages. Our results showed that egg and larval stages of Kenyan population of R1 was able to successfully complete development at 30, 33 and 35 °C, although no emergence was observed from puparia at 33 and 35 °C. The performance of the immature stages of the Kenyan population of R2 however mirrored the results obtained by Duyck and Quilici (2002). In South Africa, egg and larva of both *Ceratitis rosa* groups were able to complete development at the upper temperatures of 30, 33 and 35 °C and deviates from results obtained for the Kenya population of R2.

The reasons for these differences are unclear but nutritional elements of the diet, the biological traits of the two populations and adaptations resulting from the fact that both populations of *Ceratitis rosa* from South Africa were reared for 5–8 generations at similar experimental conditions (25 ±1 °C, 60 ± 10% RH and photoperiod of L12: D12) before the start of the experiment may have contributed to the observed variations. Indeed, populations of tephritids from different geographical regions may differ in various reproductive and life history traits (Vargas and Carey 1989, Dimantidis et al. 2011). The observed differences may contribute to the invasion potential of the different *Ceratitis rosa* populations, since population growth rates influences basic population processes that operate during invasion events (Liebhold and Tobin 2008). The results obtained also support the genetic differentiation of geographically isolated *Ceratitis rosa* populations as has been demonstrated in previous studies (Virgilio et al. 2013).

The developmental duration of the immature life stages of the two *Ceratitis rosa* groups decreased as temperature increased. This observation is consistent with earlier studies with other tephritid species (Carey et al. 1985, Fletcher 1987, Vargas et al. 1996, Vargas et al. 1997, Brèvault and Quilici 2000, Duyck and Quilici 2002, Grout and Stoltz 2007, Rwomushana et al. 2008, Salum et al. 2013, Liu and Ye 2009, Vayssières et al. 2008, Vargas et al. 2000). However, in the South African *Ceratitis rosa* populations (R1 and R2), developmental duration of the egg and larva were found to decrease with increasing temperature up to 30 °C, followed by a slight increase in developmental duration beyond 30 °C. At the larval stage, both groups of the South African *Ceratitis rosa* developed faster than the Kenyan populations. Also, the R2 from the Kenyan highland had a faster development of immature life stages at lower temperatures (15 and 20 °C) and the situation was reversed at the upper temperatures (25, 30, 33 °C) with R1 from the lowland emerging sooner than R2 from the highland. However, in the South African populations, immature larval stages of R1 tended to develop faster across all temperatures than the highland population. The larval survivorship of R1 on the artificial diet at all temperatures was significantly higher than that of R2 and could have led to higher metabolic heat within the diet and therefore faster development. To date, little is known of the selective pressures shaping the life history of immature tephritids in geographically isolated locations (Diamantidis et al. 2011a, b) and additional research focusing on the selective pressure that shape the life history of immature life stages of the different *Ceratitis rosa* populations is warranted.

For both the Kenyan and South Africa *Ceratitis rosa* populations, the values for the temperature threshold and thermal constant were not always consistent with previous studies. In La Réunion, Duyck and Quilici (2002) reported lower developmental thresholds for egg, larval and pupal stages as 9.8, 3.1, and 11.0 °C, respectively. Our lower temperature thresholds for egg and pupa are within the range reported by the previous authors. However, the estimated lower development thresholds from the linear models for larva in the Kenyan (7.1–10.3) and South African (7.8-8.6) populations are well above estimated values from La Réunion. Overall, R2 population from the highlands of Kenya tended to tolerate lower temperatures than the lowland R1 but the reverse was the case in South Africa. Duyck and Quilici (2002) reported total value of 405 DD for *Ceratitis rosa* in La Réunion. Thermal constant values for total development of the immature life stages of the Kenyan R1 and R2 were 302.75 DD and 413.53 DD, respectively. Our highland value is in agreement with data from La Réunion but differ sharply with that of the lowland population (Duyck and Quilici 2002). According to Virgilio et al. (2013) the Réunion population of *Ceratitis rosa* is referred to as R2. Therefore, the fact that our results of R2 are more in accordance with that reported by Duyck and Quilici (2002) is thus not surprising. This confirms that the highland population of R2 in Kenya might likely be of the same genotype as the Réunion population. The total thermal constant values for the South Africa population were low for the two *Ceratitis rosa* populations (R1: 342 DD, and R2: 380 DD). Fletcher (1989) noted that large differences in thermal requirements among various species of tephritids can be attributed to difference in experimental methodologies and geographic variation of populations. Besides geographic origin, factors such as food quantity and quality and larval density in the rearing chambers have been reported to influence the thermal requirements of larval stages of tephritids (Vargas et al. 1996, Duyck and Quilici 2002).

No previous studies are available in literature with regard to upper developmental threshold for *Ceratitis rosa*. However, Brière-1 nonlinear model used in this study predicted that immature stages of R1 were more tolerant to heat than R2 and this irrespective of the area of origin. Observed values clearly showed higher survivorship and faster development for R1 compared to R2 for the South African populations. In non linear models differences seemed were very small. *Ceratitis rosa* R2 did not complete development at 30 degrees. Lethal temperature values generated here may be relevant for future development regarding post harvest dis-infestation treatments for the two populations of *Ceratitis rosa*.

Both populations of *Ceratitis rosa* from Kenya and South Africa survived at temperatures of 15, 20, 25, 30 and 33 °C but no adult emerged from puparia at 10, 33 and 35 °C. In the Kenyan populations, survival of all developmental stages at temperatures other than 10 and 35 °C was > 50% which is consistent with previous studies assessing the effect of constant temperatures on development and survival of tephritids (Vargas et al. 1996). In contrast, survivorship of the South African populations was < 40% at the larval stage at the upper temperatures of 30 and 33 °C and is in agreement with earlier studies (Duyck and Quilici 2002). Overall, high survival of both populations from Kenya across a wide range of temperatures suggest that *Ceratitis rosa* from this part of the world could potentially have higher invasive powers than the South African populations and warrant careful attention in terms of monitoring and surveillance to minimize its advertent translocation and potential establishment.

## Conclusion

In conclusion, our results clearly demonstrates and support the existence of two genetically distinct populations of *Ceratitis rosa* that are divergent in their physiological response to temperature with potential consequent implications in the invasion dynamics of the pest. Difference in parameters measured between the Kenyan and South African populations may reflect certain attributes such as the diet used in the experiments, rearing procedures and adaptation processes of the insects. The findings suggest the need for taxonomic revision of *Ceratitis rosa* but additional information from integrative morphological, molecular, cytogenetic, behavioural and chemoecological data may be needed to accomplish this task.
